# Association of medication adherence with treatment preferences: incentivizing truthful self-reporting

**DOI:** 10.1007/s10198-025-01760-z

**Published:** 2025-02-22

**Authors:** Carina Oedingen, Raf van Gestel, Samare P. I. Huls, Georg Granic, Esther W. de Bekker-Grob, Jorien Veldwijk

**Affiliations:** 1 Erasmus School of Health Policy & Management, Erasmus University Rotterdam, Rotterdam, The Netherlands; 2 Erasmus Choice Modelling Centre (ECMC), Erasmus University Rotterdam, Rotterdam, The Netherlands; 3 Erasmus Centre for Health Economics Rotterdam (EsCHER), Erasmus University Rotterdam, Rotterdam, The Netherlands; 4 Erasmus School of Economics, Erasmus University Rotterdam, Rotterdam, The Netherlands; 5 Leuven Institute for Healthcare Policy, KU Leuven, Leuven, Belgium

**Keywords:** Medication adherence, Treatment preferences, Truthful self-reports, Incentivizing, Discrete choice experiment, Choice-matching

## Abstract

**Objective:**

Self-reported medication adherence may be influenced by socially desirable answers and untruthful reporting. Misreporting of adherence behavior can bias estimations of treatment (cost)effectiveness. This study investigated how to induce truthful self-reported medication adherence and evaluated how self-reported (truth-induced vs. regularly reported) medication adherence and treatment preferences were associated.

**Methods:**

Medication adherence was measured after a discrete choice experiment eliciting stated preferences for Multiple Sclerosis (MS)-treatments. Data was collected among MS-patients in three Western countries. Half of the sample was randomized to ‘choice-matching’, a novel mechanism which induces truthfulness. It financially compensates respondents based on their self-reported adherence and guesses about other respondents’ adherence. To investigate the impact of truth-incentivized adherence reporting on preference heterogeneity, interaction effects between medication adherence and treatment preferences were tested separately within the choice-matching and the ‘standard’ group.

**Results:**

The sample comprised 380 MS-patients (mean age 41y, 69% female). Respondents in the choice-matching group reported a lower medication adherence compared to the standard group (always adherent: 39.3% vs. 46.6%). Mixed logit models showed significant interaction effects: in the choice-matching group, higher medication adherence resulted in lower utility for pills twice/day compared to injections three times/week (*p* = 0.019), while in the standard group, respondents with higher medication adherence preferred pills once/day compared to injections three times/week (*p* = 0.005).

**Conclusion:**

Choice-matching likely encouraged respondents to report their true medication adherence. Linking truthful behavior to patients’ preferences allows for a better understanding of preference heterogeneity and helping to make decisions that fit patients’ true preferences.

**Supplementary Information:**

The online version contains supplementary material available at 10.1007/s10198-025-01760-z.

## Introduction

Medication adherence is defined as “the extent to which a patient acts in accordance with the prescribed interval and dose of a dosing regimen” [1]. For many patients, medication adherence is sensitive, making insights into the impact of medication adherence on treatment preferences important [2, 3]. However, low levels of medication adherence are a widespread problem for many medication therapies [4–6] and are associated with high healthcare costs worldwide [4, 7–11]. Medication adherence can be influenced by therapy-, disease-, and patient-related characteristics [8, 12]. Some of these characteristics relate to passive non-adherence behavior such as forgetting to take the medication (known as non-intentional), while other characteristics relate to active non-adherence behavior such as the conscious decision not to take the medication (known as intentional) [12]. Patients who are struggling to be adherent, may prefer a treatment option that is easier to take for instance because of a low frequency of administration or because monitoring of adherence is done by a physician (e.g., in case of injections or infusions). This relationship can also be understood the other way around, so that patients who prefer a specific treatment option may struggle less with being adherent. In summary, for patients to choose the best medication therapy requires them both to accurately assess their own medication adherence and to factor in their preferences over different treatment options. Current preference studies are usually focusing on treatment preferences without linking them to medication adherence behavior [13].

When measuring medication adherence in surveys, self-reports are a popular tool and are used in a wide range of health research [14–19]. Whereas self-reports offer an efficient and cost-effective way to collect data, it is often impossible to verify the veracity of self-reported information. It has been shown that self-reports involve the risk of systematic misreporting and response biases resulting in lower external validity of study outcomes [20, 21]. Respondents may not report truthfully either because they do not remember accurately or because they provide an answer that make them ‘look good’ [21]. In those cases, respondents tend to misreport their true behaviors and report a biased or socially desirable answer instead [22]. Thus, untruthful survey responses are a concern resulting in misreporting on the frequency of patients’ medication use and subsequently misleading the results and recommendations of economic analyses [23].

To address the problem of social desirability in self-reports on non-health related behavior, several methods have been developed and tested that financially reward truthful reporting, even when the truthfulness of answers is non-verifiable to the researchers [24–31]. One of these is choice-matching [27], a novel mechanism that is related to the Bayesian truth serum [29, 32, 33]. Choice-matching is a mechanism designed to encourage truthful answers to multiple choice questions by combining two tasks: Respondents answer the primary question (say medication adherence) and predict how others might respond to it. Respondents are rewarded financially depending on both their accuracy in predicting others and the similarity of their answers to those who chose the same response, creating a financial incentive to answer truthfully [27]. This study investigates the impact of truth-inducing incentives, using choice-matching [27] on self-reported medication (passive and active) adherence. It is subsequently investigated how truth-induced self-reports can explain heterogeneity in stated preferences. In other words, we investigate how to induce truthful self-reported medication adherence and whether this can explain heterogeneity in patients’ treatment preferences.

## Methods

### Study design

This study used a self-completed online survey containing choice-matching to induce truthful responses about self-reported medication adherence as well as a discrete choice experiment (DCE) to elicit treatment preferences. We chose treatment therapies for multiple sclerosis (MS)-patients as a case example, because optimal medication adherence is crucial to maximize treatment efficacy and reduce unnecessary healthcare expenditures [34–37]. Details on the overall study design can be found elsewhere [38].

Choice-matching has been theoretically proposed by Cvitanić et al. [27] as a mechanism to elicit truthful responses to a multiple choice question, when the truth is not verifiable. It uses two questions: (1) A ‘primary’ question asking about behaviors. This question is of direct interest. In our case, the primary question asks respondents to state their medication adherence. (2) An auxiliary question asks respondents to guess how often a certain answer to the primary question was chosen by all other respondents. In our case, respondents guess how many of ten other respondents have chosen to answer ‘Never’ to the medication adherence questions indicating that they never miss a dose of medication (i.e., fully adherent). Theoretically, choice-matching exploits the well-established empirical regularity that people in their guesses to auxiliary questions tend to overestimate the popularity of their own preferences/choices [21, 32], say medication adherence. From this regularity, it follows that people tend to *believe* that the average accuracy in guesses of all those with the same true level of medication adherence is better than the one for those respondents with a different level of true medication adherence [27]. The latter part, thus, ensures that under empirically plausible assumptions [32, 33], choice-matching provides respondents with incentives to truthfully reveal their medication adherence in the primary question [27]. In comparison to related mechanisms that financially reward truth-telling in questions of a non-verifiable truth [24–26, 28–31], choice-matching is the ‘simplest’ one [26]. Being mindful of our sample, we implemented a ‘light’ version of choice-matching to not overburden our respondents with the technical details of the procedure. In the survey, we reminded respondents to answer truthfully, that there is a financial incentive, and that their financial incentive depends on the accuracy of their guesses. That is, respondents were made to believe that there is an incentive and that this is calculated even though they do not have details of the incentive based on them being honest (detailed information about the choice-matching method can be found in the supplementary material).

DCEs are typically conducted to elicit preferences from individuals on health-related objects. It assumes that given a choice set and a hypothetical scenario, each respondent selects the alternative that maximizes their utility. Choices are behaviors that imply inequalities in utility (e.g., treatment A > treatment B) that resolve the ambiguities between objects. The higher the utility, the higher the preference for a particular object (i.e., treatment attribute) relative to other attributes and the greater the effect on the choices. A DCE was used as it allows to capture trade-offs between these attributes, revealing how respondents value different aspects of treatment.

**Fig. 1:**
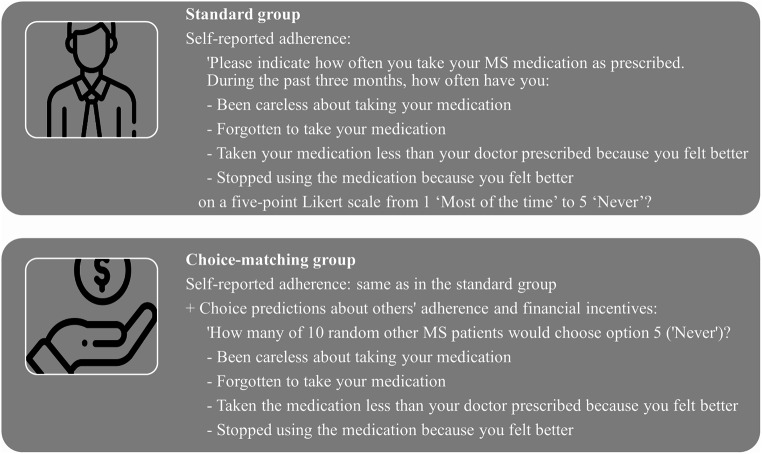
Study design

Respondents were randomly allocated to one of two groups: As can be seen in Fig. 1, respondents in the standard group were only self-reporting their medication adherence and respondents in the choice-matching group were being exposed to choice-matching as proposed by Cvitanić et al. [27].

### Questionnaire

In both groups, self-reported medication adherence was measured using a four-item scale derived from Brooks’ Medication Adherence Scale [39] by Erickson et al. [40] immediately after the DCE. Responses to the four questions were elicited using a five-point Likert scale from 1 ‘Most of the time’ to 5 ‘Never’, denoting the frequency of four different types of non-adherence during three months prior to data collection. The four questions asked how often respondents have been careless and have forgotten (e.g., passive non-adherence) using their medication, as well as have taken less and have stopped (e.g., active non-adherence) using their medication with a Likert scale indicating that a higher score means to be more adherent (detailed information about own and others’ adherence questions can be found in Fig. 1 and in the supplementary material). The total self-reported medication adherence mean score (in the following called respondents’ own adherence) was calculated by summing the responses and dividing the total sum by the four questions. Additionally, respondents in the choice-matching group were asked to guess for each of the four self-reported questions how many of ten random other MS-patients reported to be fully adherent. It was stated that a financial compensation between €1 and €5 will be donated to a national MS-foundation and that the donated amount will depend on the accuracy of the predictions in accordance with the protocol of their choice-matching procedure explained earlier. Rewarding respondents via donations to a third party induces the same truth-telling incentives as rewarding them directly if they care sufficiently about the good cause of the third party [41].

**Fig. 2:**
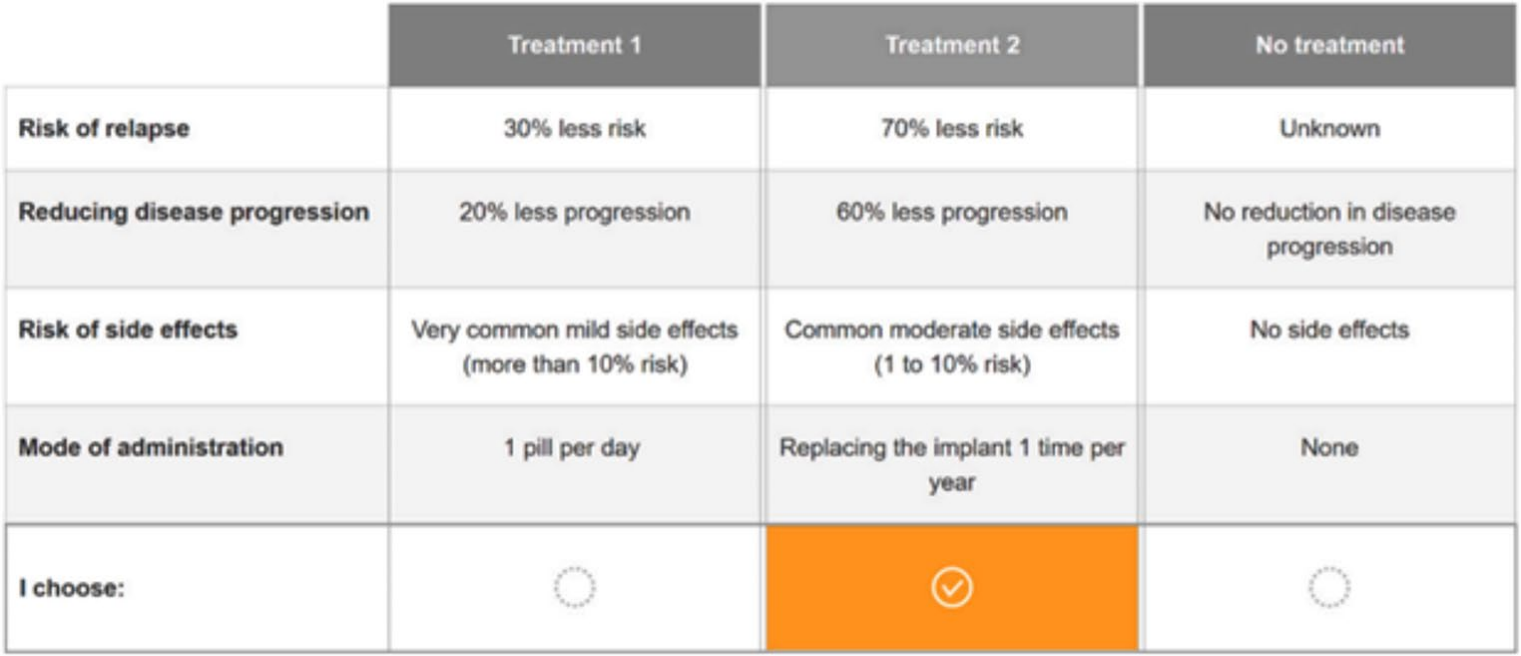
Example of a choice set

For the DCE, the following four attributes with three or six attribute levels were used: risk of relapse (30%, 50%, 70% less risk), reducing disease progression (20%, 40%, 60% less disease progression), risk of side effects (very common mild side effects: more than 10% risk, common moderate side effects: 1 to 10% risk, rare severe side effects: 0.1 to 1% risk), and mode of administration (injecting treatment once/week, injecting treatment 3 times/week, taking 1 pill/day orally, taking 2 pills/day orally, replacing the implant once/year, replacing the implant/3 years). Efficacy and safety were key themes, leading to the selection of these four attributes to capture the range of plausible outcomes for current treatments and the implant. The implant is a new mode of administration and may possible be seen as an addition to the current treatment landscape expecting that patients will face tough trade-offs concerning the mode of administration. All attributes and attribute levels were derived from systematic literature reviews and were cross-validated during two focus group discussions with MS-patients (*N* = 16) held in the Netherlands and by consulting two French MS-specialists [38]. We chose these three ranges of levels for the attributes ‘risk of relapse’, ‘reducing disease progression’ and ‘risk of side effects’ as in Europe all drugs, including disease modifying treatments, must be distributed with a comprehensive patient information leaflet (drug label) [42]. A Bayesian heterogeneous choice design was created using Ngene ChoiceMetrics software [43] to maximize D-efficiency [44]. The final experimental design contained 30 choice sets that were divided into two blocks of 15 choice sets. Each choice set had two unlabeled alternatives (‘treatment 1’ and ‘treatment 2’) that were characterized by a selection of attribute levels, and the third alternative (‘no treatment’) allowed respondents, like in real-life, to not choose any of the alternatives presented (i.e., opt-out) [38]. An example of a choice set can be found in Fig. 2, while a list of the attributes and attribute levels as well as the experimental design are shown in the supplementary material.

In addition, the questionnaire contained questions about respondent’s demographics, health status, numeracy skills [45, 46], health literacy [47, 48] and health-risk attitude items [49]. Finally, six concluding questions about perceived difficulty and length of the questionnaire were also included. The questionnaire was pre-tested using the think-aloud method in four Dutch MS-patients and pilot-tested with *n* = 100 respondents. After data collection was completed in the Netherlands, the questionnaire was back and forward translated into English (by the research team) and French (by a translation company). Throughout the whole questionnaire, there were no back buttons, so respondents were not able to revise their earlier responses [38].

### Data collection and study sample

Respondents were recruited through the commercial survey sampling company SurveyEngine in three Western European countries (the Netherlands, France and the United Kingdom). In addition, patients were also recruited via national patient advocacy groups. Respondents were included when they were at least 18 years old, diagnosed with MS, living in the Netherlands, France or the United Kingdom, speaking and understanding Dutch, French or English and giving written informed consent. We excluded *n* = 8 respondents with missing data for the choice-matching. Careful testing showed that there was nothing systematic about this small group. All respondents received financial compensation (less than €5) upon completion of the questionnaire (this was on top of the donation to a national MS-foundation for the choice-matching group). The study was approved by the Medical Ethical Testing Committee of the Erasmus Medical Centre (vote number: MEC-2019-0248) [38].

### Statistical analysis

Stata (StataCorp LLC MP 18.0) was used for all statistical analyses. Baseline characteristics were calculated as means with standard deviations for continuous variables and absolute numbers with percentages for categorical variables. Differences between respondents of the standard and choice-matching group were analyzed with the Kruskal-Wallis test and Wilcoxon signed-rank test for the variables measured on a continuous scale and with chi-squared tests for the variables measured on a discrete scale. Furthermore, we compared the median Likert responses for each question about own adherence and the mean scale as well as the median Likert responses for each question of choice predictions about others’ adherence and the mean scale between groups [50, 51].

To determine the influence of choice-matching on self-reported medication adherence and to assess whether truthful medication adherence explains preference heterogeneity, mixed logit models (MXL) with 1,000 Halton draws were conducted for both groups separately. With MXL models, we account for the use of panel data (i.e., that each respondent completed 15 choice sets) and are able to investigate heterogeneity of preferences. The MXL model identifies attributes for which there is significant preference variation strictly based on variations in choice patterns across individuals. Including interaction effects between the attribute ‘mode of administration (implants, pills or injections)’ and respondents’ own adherence allows for testing whether respondents with higher medication adherence have different preferences for this attribute compared to respondents with a lower medication adherence. First, we calculated an overall MXL model including interaction effects between respondents’ own adherence (a mean score derived from all four questions measuring self-reported medication adherence) and ‘mode of administration’. Second, we calculated two MXL models differentiating between passive and active respondents’ own adherence. A passive adherence includes the two items about ‘being careless’ and ‘having forgotten’ using the medication, while an active adherence is described by the other two items about ‘taking less’ and ‘having stopped’ using the medication. Furthermore, relative attribute importance was calculated based on the overall MXL model. Statistical significance indicates whether a level influenced the respondents’ choice. The results are expressed as parameter estimates (β), their 95% confidence interval (CI), and p-values. We used dummy-coding for the attribute levels, which were entered as explanatory variables in the models and continuous-coding for the self-reported own medication adherence.

## Results

### Study characteristics

Overall, 380 respondents (standard group *n* = 189; choice-matching group *n* = 191) completed the survey. They had a mean age of 41 years old, more than two thirds were female (*n* = 264), 51% (*n* = 195) of the respondents had a relapsing-remitting MS and were able to walk without an aid (*n* = 187, 49%). Generally, respondents found the survey easy (strongly agree: *n* = 192, 51%; *n* = 86, somewhat agree: 23%) and one third could have answered more questions (strongly agree: *n* = 145, 38%; *n* = 91, somewhat agree: 24%), which resulted in a mean response time of 17 min for the whole survey. There were no significant differences between the groups in the socio-demographic characteristics except for a slightly lower respondents’ age in the choice-matching group (Table 1).

**Table 1:** Descriptive statistics for the samples

Variable	Definition	Standard group (*n* = 189)	Choice-matching group (*n* = 191)	*p*-value
*Socio-demographic characteristics*				
Age in years, mean (SD)		42.40 (± 12.33)	39.79 (± 12.04)	0.012**
Sex, n (%)	Female	132 (69.84)	132 (69.11)	0.877
	Male	57 (30.16)	59 (30.89)	
Nationality, n (%)	Dutch	56 (29.63)	59 (30.89)	0.963
	French	63 (33.33)	62 (32.46)	
	British	70 (37.04)	70 (36.65)	
Education^a^, n (%)	Primary	9 (4.76)	8 (4.19)	0.245
	Secondary	42 (22.22)	30 (15.71)	
	Vocational/technical	40 (21.16)	55 (28.80)	
	University	97 (51.32)	95 (49.74)	
*MS-disease*				
Age diagnosed with MS in years, mean (SD)		31.14 (± 11.73)	28.97 (± 11.65)	0.100
Disease progression^b^, n (%)	Clinically isolated syndrome	25 (13.23)	17 (8.90)	0.235
	Relapsing-remitting MS	100 (52.91)	95 (49.74)	
	Primary progressive MS	36 (19.05)	36 (18.85)	
	Secondary progressive MS	21 (11.11)	28 (14.66)	
Current mobility, n (%)	Able to walk without an aid, and have no, mild or moderate MS-symptoms	93 (49.211)	94 (49.21)	0.867
	Able to walk with an aid (for example, up to and including 20–300 m), or have to use a wheelchair to be mobile	90 (47.62)	89 (46.60)	
	Restricted to bed or pushed in a wheelchair	6 (3.17)	8 (4.19)	
*Understanding health information and survey comprehension*				
How easy is it for you to understand the obtained information, mean (SD)	Response to the statement on a Likert scale from 1 (“very easy”) to 4 (“very difficult”)	2.16 (± 0.81)	2.13 (± 0.64)	0.918
How easy is it for you to apply the obtained information to your daily life, mean (SD)	Response to the statement on a Likert scale from 1 (“very easy”) to 4 (“very difficult”)	2.32 (± 0.77)	2.16 (± 0.76)	0.063
The survey was easy, mean (SD)	Response to the statement on a Likert scale from 1 (“strongly agree”) to 5 (“strongly disagree”)	1.87 (± 1.09)	1.97 (± 1.18)	0.507
I could have answered more questions, mean (SD)	Response to the on a Likert scale from 1 (“strongly agree”) to 5 (“strongly disagree”)	2.17 (± 1.29)	2.37 (± 1.31)	0.100
Response time, mean (SD)	Response time for the whole survey in minutes	16.16 (± 9.50)	18.31 (± 12.34)	0.274
Blocks, n (%)	Block 1	103 (54.50)	89 (46.60)	0.124
	Block 2	86 (45.50)	102 (53.40)	

Notes: p-values are for the null hypothesis of no difference between the two samples with respect to a given variable. a: *n* = 4 respondents reported other education. b: *n* = 22 respondents reported that they do not know their disease progression

***, **, * imply significance less then at the 0.1%, 1% and 5% level

### Self-reported medication adherence

**Fig. 3:**
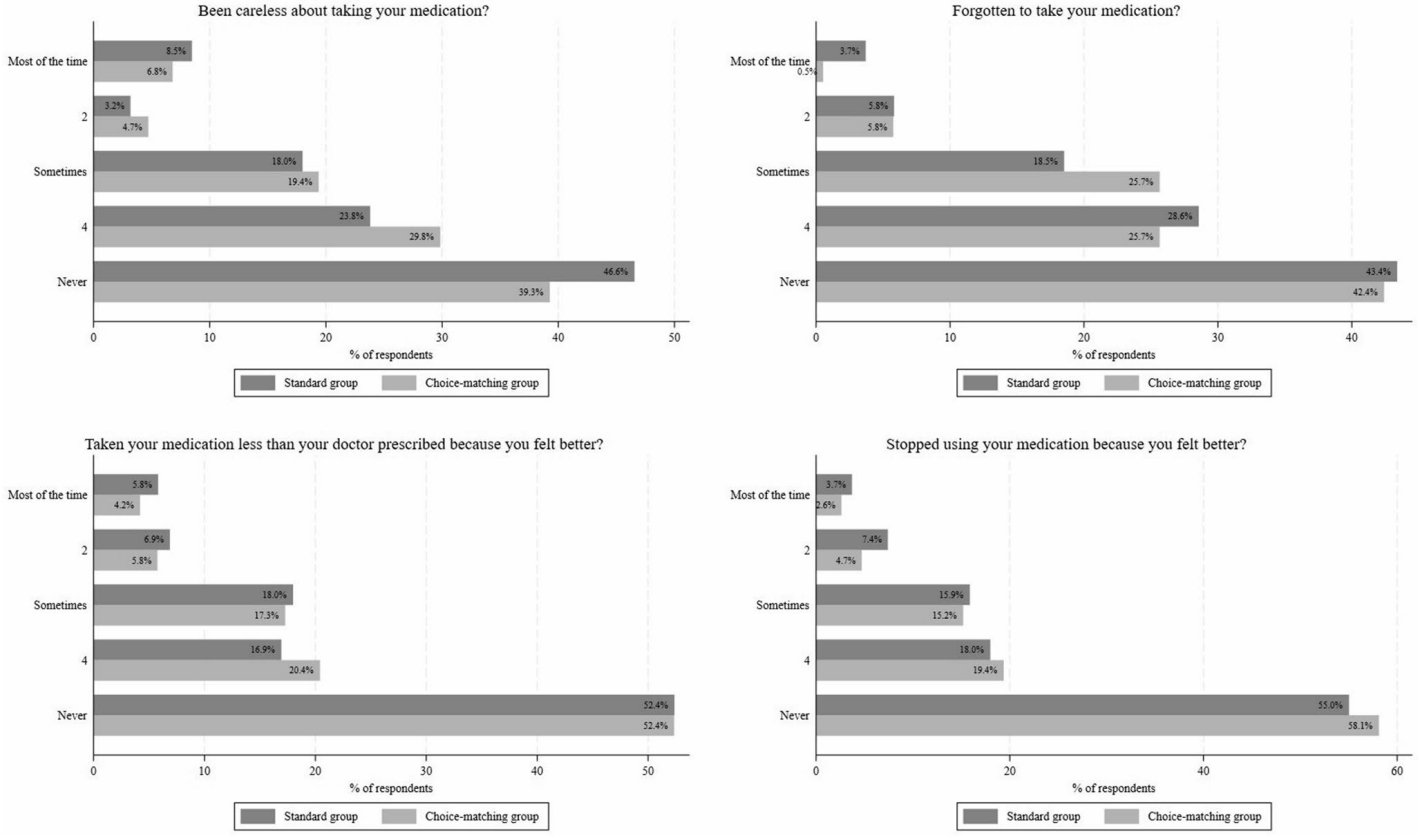
Frequencies of respondents’ own medication adherence for standard group and choice-matching group. (Note: Respondents’ own medication adherence is a mean score from 1 ‘Most of the time’ to 5 ‘Never’ indicating that a higher score means to be more adherent.)

The mean score of respondents’ own adherence did not differ significantly between the two groups (*p* = 0.938; standard group: 4.04 ± 0.979: choice-matching group: 4.08 ± 0.901). However, there are differences within the four scales measuring ‘own adherence’ (Fig. 3). In the choice-matching group, fewer respondents reported to never ‘being careless’ about taking their medication compared to the standard group (standard group: n = 88, 46.6%; choice-matching group: n = 75, 39.3%). For ‘have forgotten to take their medication’ and taking their ‘medication less than the doctors prescribed’ we did not observe differences, while more respondents from the choice-matching group stated to ‘have never stopped their medication’ (standard group: n = 104, 55.0%; choice-matching group: n = 111, 58.1%). Overall, we observed fewer responses in the extreme category of being fully adherent in the choice-matching group. Detailed information can be found in the supplementary material.

### Explaining heterogeneity in treatment preferences

The attributes ‘risk of relapse’, ‘disease progression’ and ‘mode of administration’ significantly impacted choice in both standard and choice-matching group (*p* < 0.05). In both groups, the negative constant for the opt-out showed a disutility for no-treatment over treatment, indicating that any treatment was preferred over no treatment, all else being equal. However, the coefficient was not statistically significant in the choice-matching group. For the standard deviations, all levels were significantly different from zero and had p-values of < 0.001, which revealed significant preference heterogeneity among the respondents in both groups.

**Fig. 4:**
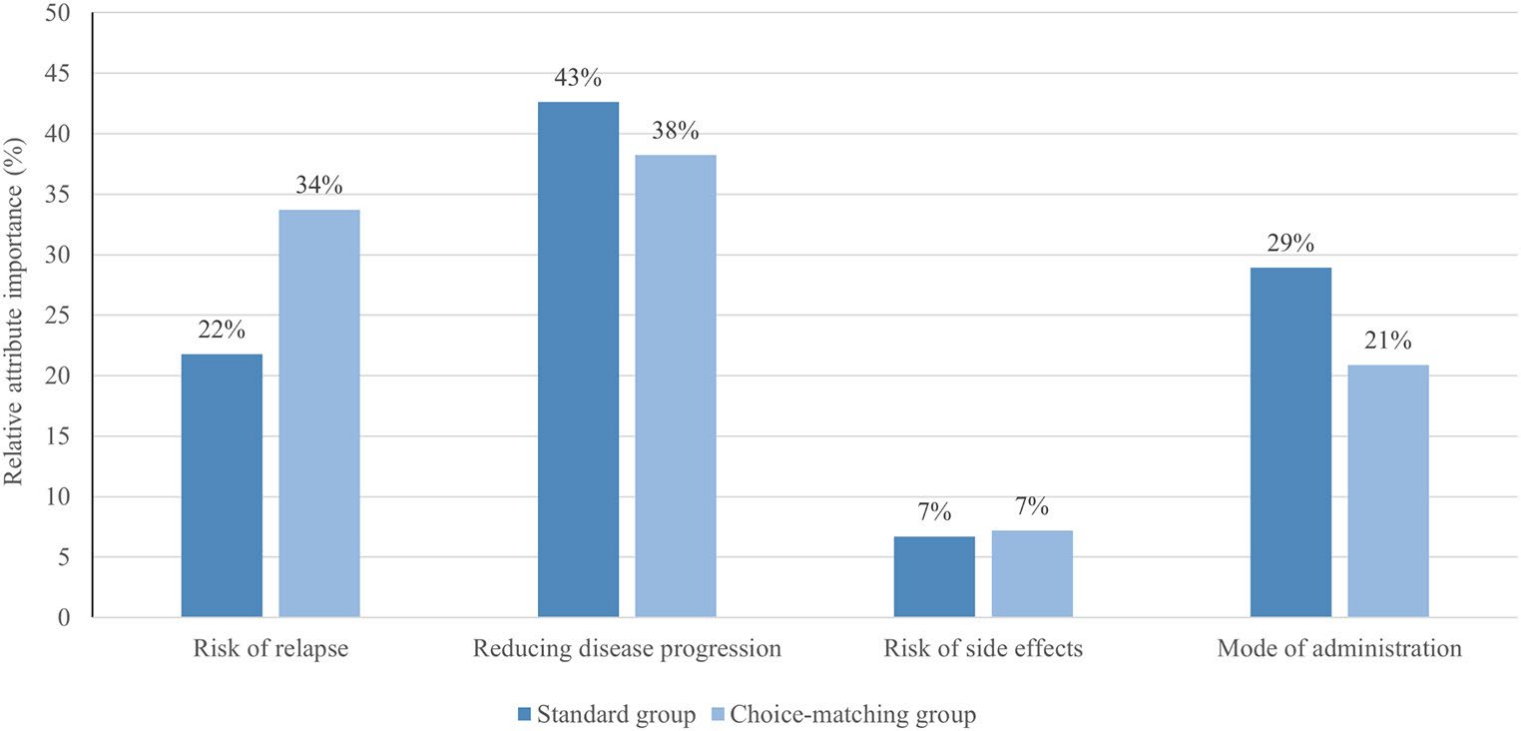
Relative attribute importance based on mixed logit estimates with random effects

Fig. 4 shows the relative attribute importance for both groups. Relative to the other attributes, ‘reducing disease progression’ (43%) and ‘mode of administration’ (29%) were the two most important attributes, while ‘risk of relapse’ (22%) was less important for respondents in the standard group. Contrary, for respondents in the choice-matching group the attributes ‘reducing disease progression’ (38%) and ‘risk of relapse’ (34%) were almost equally the most important ones, and ‘mode of administration’ was less important (21%). In both groups, ‘risk of side effects’ was the least important attribute (7%).

**Fig. 5:**
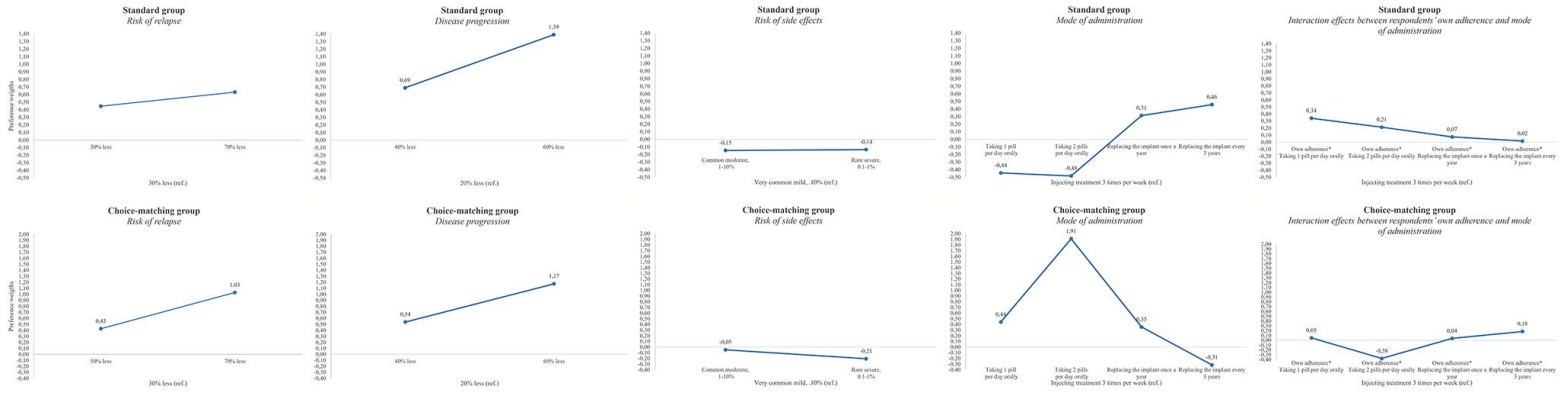
Mixed logit estimates with interaction effects between respondents’ own adherence and mode of administration

Significant interaction effects were found between self-reported medication adherence and preferences for treatment modality (see Fig. 5). In the choice-matching group, respondents with higher self-reported own adherence had a lower preference for pills twice/day compared to injections three times/week (*p* = 0.019) compared to respondents with a lower self-reported own adherence in this group. In the standard group, respondents with a higher self-reported own adherence showed a utility for taking pills once/day compared to injections three times/week (*p* = 0.005) compared to respondents with a lower self-reported own adherence in this group.

When differentiating between respondents’ own passive (see Table 2A) and active (see Table 2B) adherence, we observed the same results as in the overall respondents’ own adherence: In the choice-matching group, respondents with higher own passive adherence had a lower preference for pills twice/day compared to injections three times/week (*p* = 0.043) compared to respondents with a lower adherence in this group. In the standard group, respondents with higher own adherence preferred pills once/day compared to injections three times/week (*p* = 0.022) compared to respondents with a lower adherence in this group. Regarding own active adherence, we only observed differences for the standard group, and not for the choice matching group: Respondents with higher own active adherence showed higher utility scores not only for pills once/day compared to injections three times/week (*p* = 0.002), but also for injections once/week compared to injections three times/week (*p* = 0.027) compared to respondents with a lower adherence in this group.

**Table 2:** Mixed logit estimates with interaction effects between respondents’ own passive and active adherence and mode of administration A: respondents’ own passive adherence and mode of administration

	Standard group					Choice-matching group				
Attributes	Coef	SE	95% CI		*p*-value	Coef	SE	95% CI		*p*-value
*Mean preference weights*										
Risk of relapse (ref: 30% less)										
50% less	0.437	0.107	0.227	0.647	**0.001**	0.427	0.117	0.198	0.656	**0.001**
70% less	0.644	0.121	0.406	0.881	**0.001**	1.029	0.137	0.760	1.298	**0.001**
Disease progression (ref: 20% less)										
40% less	0.662	0.114	0.439	0.885	**0.001**	0.536	0.114	0.312	0.760	**0.001**
60% less	1.354	0.158	1.045	1.662	**0.001**	1.174	0.158	0.863	1.484	**0.001**
Risk of side effects (ref: Very common mild, > 10%)										
Common moderate, 1–10%	-0.124	0.114	-0.348	0.100	0.279	-0.048	0.115	-0.272	0.177	0.678
Rare severe, 0.1-1%	-0.146	0.115	-0.371	0.079	0.202	-0.209	0.129	-0.461	0.044	0.106
Mode of administration (ref: Injecting treatment 3 times per week)										
Injecting treatment once a week	-0.219	0.510	-1.219	0.781	0.668	0.436	0.643	-0.825	1.696	0.498
Taking 1 pill per day orally	-0.153	0.478	-1.090	0.783	0.749	0.466	0.569	-0.648	1.581	0.412
Taking 2 pills per day orally	-0.160	0.511	-1.160	0.841	0.755	1.594	0.623	0.373	2.816	**0.011**
Replacing the implant once a year	0.288	0.504	-0.700	1.277	0.568	0.617	0.553	-0.467	1.701	0.265
Replacing the implant every 3 years	0.130	0.499	-0.848	1.109	0.794	0.419	0.573	-0.705	1.543	0.465
*Interaction effects (ref: Injecting treatment 3 times per week)*										
Own adherence*Injecting treatment once a week	0.072	0.123	-0.169	0.314	0.558	-0.115	0.158	-0.424	0.194	0.465
Own adherence*Taking 1 pill per day orally	0.264	0.115	0.039	0.489	**0.022**	0.046	0.140	-0.228	0.321	0.741
Own adherence*Taking 2 pills per day orally	0.131	0.122	-0.109	0.371	0.284	-0.309	0.152	-0.607	-0.010	**0.043**
Own adherence*Replacing the implant once a year	0.084	0.122	-0.156	0.324	0.493	-0.029	0.134	-0.292	0.234	0.829
Own adherence*Replacing the implant every 3 years	0.092	0.120	-0.142	0.326	0.442	0.005	0.138	-0.266	0.276	0.970
*Alternative-specific constant*	-0.318	0.131	-0.574	-0.061	**0.015**	-0.092	0.130	-0.347	0.163	0.481
*Standard deviation of preference weights*										
Risk of relapse (ref: 30% less)										
50% less	-0.830	0.132	-1.089	-0.571	**0.001**	1.056	0.122	0.817	1.294	**0.001**
70% less	1.115	0.136	0.848	1.381	**0.001**	1.344	0.134	1.082	1.607	**0.001**
Disease progression (ref: 20% less)										
40% less	0.957	0.132	0.699	1.215	**0.001**	0.955	0.134	0.693	1.217	**0.001**
60% less	1.546	0.143	1.265	1.826	**0.001**	1.556	0.146	1.270	1.842	**0.001**
Risk of side effects (ref: Very common mild, > 10%)										
Common moderate, 1–10%	0.804	0.131	0.546	1.061	**0.001**	0.718	0.131	0.461	0.975	**0.001**
Rare severe, 0.1-1%	0.966	0.124	0.724	1.208	**0.001**	1.158	0.124	0.914	1.401	**0.001**
Mode of administration (ref: Injecting treatment 3 times per week)										
Injecting treatment once a week	0.699	0.249	0.211	1.188	**0.005**	1.184	0.206	0.780	1.588	**0.001**
Taking 1 pill per day orally	0.919	0.150	0.625	1.214	**0.001**	-1.096	0.148	-1.386	-0.806	**0.001**
Taking 2 pills per day orally	1.067	0.169	0.736	1.398	**0.001**	1.311	0.170	0.977	1.645	**0.001**
Replacing the implant once a year	0.954	0.190	0.582	1.326	**0.001**	-0.900	0.191	-1.274	-0.525	**0.001**
Replacing the implant every 3 years	-0.896	0.184	-1.256	-0.536	**0.001**	1.019	0.180	0.665	1.372	**0.001**
LL (null)	-2647.741					-2767.095				
LL (model)	-2418.199					-2481.535				
AIC	4892.398					5019.07				
BIC	5089.754					5216.721				
N	189					191				
Observations	8,505					8,595				

Notes: Passive adherence includes the items been careless about taking and forgotten to take your medication; AIC = Akaike information criterion; BIC = Bayesian information criteria; CI = confidence interval; Coef = coefficient; LL = log likelihood; SE = standard error; p-value are bold < 0.05

**Table 2:** Mixed logit estimates with interaction effects between respondents’ own passive and active adherence and mode of administration B: respondents’ own active adherence and mode of administration

	Standard group					Choice-matching group				
Attributes	Coef	SE	95% CI		*p*-value	Coef	SE	95% CI		*p*-value
*Mean preference weights*										
Risk of relapse (ref: 30% less)										
50% less	0.404	0.111	0.186	0.622	**0.001**	0.465	0.121	0.229	0.701	**0.001**
70% less	0.622	0.128	0.372	0.872	**0.001**	1.002	0.137	0.735	1.270	**0.001**
Disease progression (ref: 20% less)										
40% less	0.658	0.115	0.433	0.882	**0.001**	0.567	0.115	0.342	0.793	**0.001**
60% less	1.335	0.156	1.029	1.642	**0.001**	1.123	0.155	0.818	1.428	**0.001**
Risk of side effects (ref: Very common mild, > 10%)										
Common moderate, 1–10%	-0.145	0.115	-0.370	0.079	0.205	-0.066	0.111	-0.283	0.151	0.511
Rare severe, 0.1-1%	-0.152	0.116	-0.380	0.076	0.192	-0.182	0.127	-0.430	0.066	0.150
Mode of administration (ref: Injecting treatment 3 times per week)										
Injecting treatment once a week	-1.062	0.545	-2.130	0.006	0.051	-0.460	0.589	-1.615	0.695	0.435
Taking 1 pill per day orally	-0.467	0.469	-1.287	0.453	0.320	0.837	0.497	-0.137	1.810	0.092
Taking 2 pills per day orally	-0.386	0.515	-1.394	0.623	0.453	1.580	0.561	0.480	2.681	**0.005**
Replacing the implant once a year	0.294	0.504	-0.693	1.281	0.559	0.252	0.543	-0.812	1.316	0.643
Replacing the implant every 3 years	0.390	0.514	-0.618	1.397	0.448	-0.589	0.560	-1.687	0.509	0.293
*Interaction effects (ref: Injecting treatment 3 times per week)*										
Own adherence*Injecting treatment once a week	0.285	0.129	0.032	0.539	**0.027**	0.095	0.137	-0.174	0.364	0.487
Own adherence*Taking 1 pill per day orally	0.343	0.111	0.126	0.560	**0.002**	-0.032	0.117	-0.261	0.196	0.782
Own adherence*Taking 2 pills per day orally	0.187	0.122	-0.052	0.426	0.126	-0.281	0.133	-0.542	-0.020	**0.035**
Own adherence*Replacing the implant once a year	0.081	0.119	-0.153	0.315	0.498	0.068	0.125	-0.177	0.314	0.586
Own adherence*Replacing the implant every 3 years	0.038	0.121	-0.198	0.275	0.752	0.253	0.130	-0.001	0.507	0.051
*Alternative-specific constant*	-0.349	0.132	-0.607	-0.091	**0.008**	-0.107	0.130	-0.361	0.148	0.411
*Standard deviation of preference weights*										
Risk of relapse (ref: 30% less)										
50% less	-0.898	0.131	-1.154	-0.641	**0.001**	1.105	0.131	0.848	1.362	**0.001**
70% less	1.175	0.132	0.918	1.433	**0.001**	1.465	0.159	1.154	1.777	**0.001**
Disease progression (ref: 20% less)										
40% less	0.984	0.131	0.727	1.242	**0.001**	1.008	0.132	0.751	1.266	**0.001**
60% less	1.621	0.161	1.306	1.936	**0.001**	1.649	0.154	1.347	1.952	**0.001**
Risk of side effects (ref: Very common mild, > 10%)										
Common moderate, 1–10%	0.798	0.139	0.526	1.071	**0.001**	0.574	0.159	0.262	0.886	**0.001**
Rare severe, 0.1-1%	0.990	0.122	0.752	1.229	**0.001**	1.190	0.129	0.937	1.443	**0.001**
Mode of administration (ref: Injecting treatment 3 times per week)										
Injecting treatment once a week	-0.908	0.207	-1.315	-0.502	**0.001**	-1.015	0.227	-1.459	-0.571	**0.001**
Taking 1 pill per day orally	0.880	0.147	0.592	1.168	**0.001**	0.998	0.161	0.682	1.314	**0.001**
Taking 2 pills per day orally	1.050	0.177	0.703	1.398	**0.001**	1.333	0.185	0.971	1.695	**0.001**
Replacing the implant once a year	0.874	0.183	0.516	1.232	**0.001**	-0.780	0.212	-1.195	-0.365	**0.001**
Replacing the implant every 3 years	0.923	0.166	0.599	1.248	**0.001**	0.940	0.206	0.537	1.343	**0.001**
LL (null)	-2642.35					-2759.604				
LL (model)	-2408.49					2489.719				
AIC	4872.98					5035.437				
BIC	5070.336					5233.088				
N	189					191				
Observations	8,505					8,595				

Notes: Active adherence includes the items been careless about taken your medication less than your doctor prescribed and stopped using your medication because you felt better; AIC = Akaike information criterion; BIC = Bayesian information criteria; CI = confidence interval; Coef = coefficient; LL = log likelihood; SE = standard error; p-value are bold < 0.05

## Discussion

This study aimed to investigate the impact of self-reported medication adherence on treatment preferences, the mechanism of choice-matching to induce truthful self-reported medication adherence and whether this can explain heterogeneity in MS-treatment preferences among patients. Overall, the mean score of respondents’ own adherence did not differ significantly between respondents in the choice-matching and standard group, however, fewer respondents in the choice-matching group reported to never ‘being careless’ taking their medication compared to respondents in the standard group. Those who were induced to be truthful due to the ‘financial incentive’ set by the choice-matching mechanism and reported to be more adherent, have a lower utility for pills twice/day compared to injections three times/week compared to respondents who reported to be less adherent in the same group. This finding contrasts with the standard group that reported to be more adherent, possibly untruthful, in which there is a higher preference for one pill/day compared to three injections/week compared to respondents who reported to be less adherent in the same group.

To our knowledge, this is the first study in the health domain using the choice-matching mechanism to induce truthful self-reporting. So far, no research has focused on the truthful reporting of medication adherence using the choice-matching mechanism. Previous research has been conducted in environmental economics [51] as well as in agri-food economics [52]. Both studies found that choice-matching indeed offers a method for incentivizing truthful stated preference responses.

It is interesting to note that we could not find statistically significant differences regarding the self-reported medication adherence between the two groups. This result is surprising as we expected that the choice-matching group would report to be less adherent than the standard group because of the truth-incentivized reporting. Several explanations might underly this observation. First, the case study was conducted among MS-patients, who generally already show a high adherence to their medication compared to other patients such as with Type 2 diabetes [53] or cardiovascular diseases [7]. Second, there are no treatments available to cure MS, medication adherence is already crucial for patients to reduce at least disease progression [54, 55]. Third, the descriptive statistics showed that higher educated patients are overrepresented. Systematic reviews in various disease areas have shown that higher educated patients have better abilities to adhere to certain medication regimes [12, 56, 57]. Fourth, the ‘incentive’ might have been the cause. In the choice-matching group, we made them believe to use a donation between €1 and €5 to a national MS-foundation as a financial incentive. Using donations has a practical advantage as it reduces the required amount of reward-related compensation transaction to one bulk donation, whereas rewarding respondents individually would require many micro transactions. However, the choice-matching group might not have been incentive-compatible enough to report their medication adherence truthfully because of a too low chosen compensation or an indirect donation to a charity (to which they might not care sufficiently enough) rather than direct to them [58].

Higher self-reported medication adherence in the standard group led to take pills over injections, probably because they are confident to manage their treatment. Contrary to expectations, we observed that higher self-reported medication adherence in the choice-matching group led to a disutility to take two pills over injections three times/week. So, patients who are induced to be truthful about their medication adherence, choose for a treatment option (in this case injections) that can help them to improve their adherence. A possible, but indirect, explanation for this finding could be that those who self-reported to be more adherent in the choice-matching group also are those who put a higher value on medication adherence. Injections and implants can be seen as modes of administration helping patients to overcome the full burden of responsibility as they are administered by their physicians. Therefore, patients do not need to take their medication actively every day. It can help especially those who are struggling to be adherent with the usual treatment options such as oral pills. A further possible explanation could be that for respondents in the choice-matching group the effectiveness of a treatment is more important when choosing a treatment, and less the mode of administration as shown by the relative attribute importance. We performed additional robust checks using mixed logit models with random and fixed effects supporting our observations. Interestingly, systematic reviews and meta-analyses identified higher adherence rates for oral therapies [34, 37] and previous patients’ preference studies found that MS-patients strongly preferred oral therapy [59–63] lacking the discussion of truthful reporting of medication adherence. Therefore, oral treatment can still be seen as the first treatment choice when diagnosed with MS [55]. It might also explain why the standard group reported a preference for this route of administration.

Altogether, patients’ preferences are an important component of care for MS and the involvement of patients in shared decision-making is central to improve medication adherence. Furthermore, more preferred therapies can likely be associated with increased adherence stating the relevance of medication adherence. A better understanding of patients’ preferences about their medication adherence impacting their treatment choices can influence the (cost)effectiveness of treatment, with increased adherence demonstrating significant health and economic benefits compared to non-adherence [64]. Based on our results, choice-matching seems to have (small) effects on responses, while being more resource-intensive for both researchers and respondents. Therefore, research is needed regarding incentive-induced methods as well as the chosen amount to respondents directly or indirectly via a third party. This will also help to answer to which extent researchers should induce truthfulness of respondents in stated preference research.

### Strengths and limitations

To our knowledge, we were the first to investigate the choice-matching mechanism and whether untruthful self-reports can explain heterogeneity in treatment preferences. We followed good research practices for both choice-matching and DCE [38]. Furthermore, respondents from three different countries were included in the study. However, this study has also some limitations. First, MS-diagnosis was self-reported as recruitment went through an online panel and national patient advocacy groups. Some respondents reported to have more than one rare condition (e.g., MS and Alzheimer’s disease), which might indicate fraudulent responses. Sensitivity analysis excluding these respondents (*n* = 74) from the analysis did not change the overall results confirming the robustness of our results (data not shown). Second, half of the sample had a university degree resulting in an overrepresentation of higher educated patients. Third, we only conducted focus group discussions in the Netherlands, and we did not consult with MS-specialists from the United Kingdom due to practical constraints. Furthermore, the pre-test was based on four Dutch MS-patients and the priors for all countries were based on the pilot performed in the Netherlands. To enhance comparability of the survey results, the priors were identical for all three countries. Therefore, it is possible that there is a potential bias in attribute selection. However, we found no differences in preference structures by country of residence, suggesting the validity of the DCE. As such, we do not think these choices had a great effect. Fourth, we were not able to identify differences in preferences or data quality between respondents recruited from the panel and those recruited from patient advocacy groups due to a fully anonymized data collection. And finally, the sample may be underpowered, however, performing robust checks led to similar results.

## Conclusions

We presented an empirical application of the choice-matching mechanism for truthful medication adherence reporting and investigated whether medication adherence influences treatment preferences using a case study among MS-patients. It was shown that patients who are induced to be truthful about their own medication adherence showed other treatment preferences compared to patients who are not induced to be truthful about their own adherence behavior. Linking truthful self-reporting of adherence to treatment preferences allows for a better understanding of preference heterogeneity, which in turn improves shared decision-making of treatments between patients and physicians that also fits the patients’ true treatment preferences. Future research is needed about the design of the financial incentive as well as how invasive researchers should be in inducing truthfulness of respondents.

## Electronic supplementary material

Below is the link to the electronic supplementary material.

Supplementary Material 1

## Data Availability

The datasets generated during and/or analyzed during the current study are available from the corresponding author on reasonable request. The data are not publicly available due to privacy restrictions.

## Code availability

The code generated during the current study are available from the corresponding author.
