# Supplementary material for: Association of medication adherence with treatment preferences: incentivizing truthful self-reporting
Source: Eur J Health Econ. 2025 Feb 22;26(7):1219–32. doi: 10.1007/s10198-025-01760-z (PMC12432051; doi:10.1007/s10198-025-01760-z)
Supplement: Supplementary file 1 — Supplementary Material 1 [file 10198_2025_1760_MOESM1_ESM.docx]

**Supplementary material (for web publication only)**

Association of medication adherence with treatment preferences: Incentivizing truthful self-reporting

1. **Theoretical description of choice-matching**

Choice-matching by Cvitanić et al. [27] is a theoretical proposed mechanism to elicit honest responses through economic incentives and decision theory when the truth is not verifiable. It contributes to understanding how economic mechanisms can be designed to mitigate dishonesty and promote integrity in decision-making contexts, thereby encouraging individuals to act honestly to match their stated intentions or commitments.

Choice-matching relies on the use of two questions: (1) one question asks about opinions, judgements, or preferences; and (2) based on this information an auxiliary question is employed to determine an actual outcome (this can be any question but it has to affect a participant in some way, for example, through payoffs) based on the answers of other individuals of the same type. The main idea behind the incentivization is that those who think alike in the first question (that is, those of the same type) give similar answers to the auxiliary question, because the two questions are designed to be thematically connected. An individual wants the actual outcome to be determined by the own response to the auxiliary question. If the outcome is instead determined by the responses of others, it is desirable that these others are of the same type. This is because they are more likely to give similarly answers to the auxiliary question than the different-type individuals. This implies that it pays off to reveal own true opinions, judgements or preferences in order to be assigned to the right (true) type.

The method is related to the Bayesian truth serum (BTS) by Prelec [29], however, choice-matching is administered differently through a survey and analyzed differently from the BTS method. BTS calculates a score which consists of two components: i) a prediction score that reward individuals’ predictions based on their accuracy with respect to the others’ choice behavior, and ii) an information score that rewards individuals’ personal choices based on whether these are surprisingly common, meaning these choices are more frequent than predicted.

1. **Questions about own and others’ medication adherence**

**Self-reported medication adherence questions (both standard and choice-matching group)**

*Please indicate how often you take your MS medication as prescribed. If you are currently not taking MS medication, please indicate how often you take other medication as prescribed.*

*During the past three months, how often have you:*

Select one answer per row.

|  | 1  Most of the time | 2 | 3  Sometimes | 4 | 5  Never |
| --- | --- | --- | --- | --- | --- |
| Been careless about taking your medication? |  |  |  |  |  |
| Forgotten to take your medication? |  |  |  |  |  |
| Taken your medication less than your doctor prescribed because you felt better? |  |  |  |  |  |
| Stopped using your medication because you felt better? |  |  |  |  |  |

**Others’ medication adherence questions (choice-matching group only)**

*Please indicate how often you take your MS medication as prescribed. If you are currently not taking MS medication, please indicate how often you take other medication as prescribed. You will first be asked to relate the question to yourself, then you will be asked to indicate how you think others will answer it. Please answer the question carefully and to the best of your knowledge. Depending on how you fill out the questions, a financial compensation between €1 and €5 will be donated to a national MS foundation. The amount donated will depend on how accurate your prediction was. How many of 10 random other MS patients would choose option 5 ('Never') for the statements below?*

**Example of the survey for standard group**


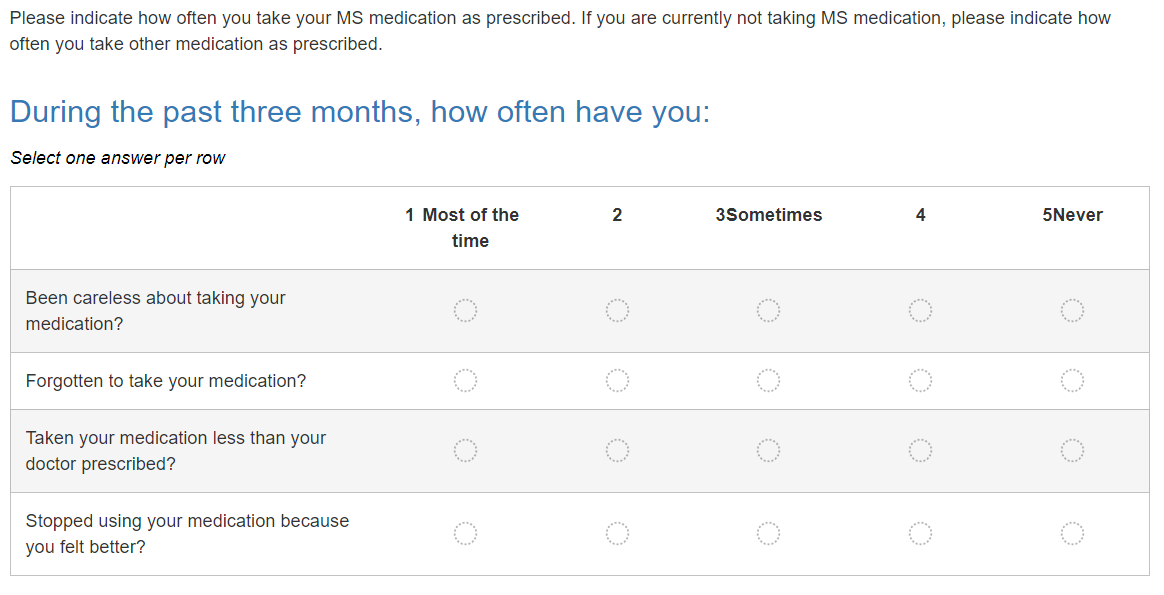


**Example of the survey for choice-matching group**


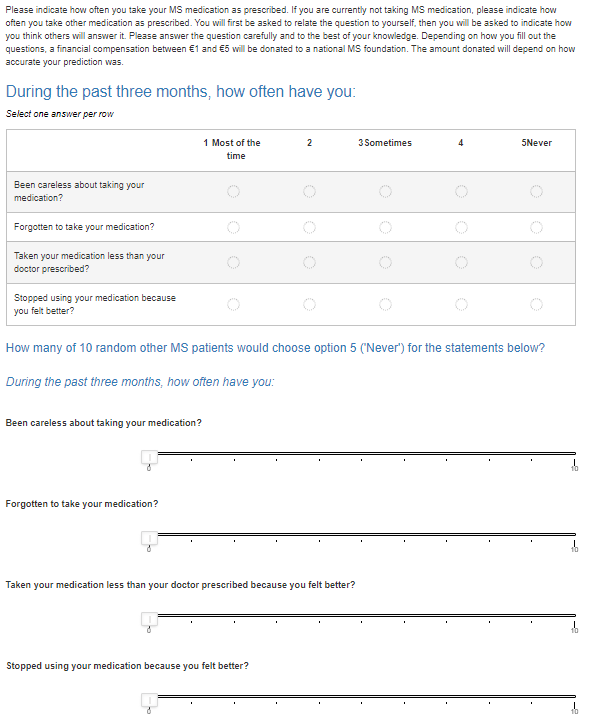


1. **List of attributes and attribute levels from the DCE [38] and the experimental design**

| **Attribute** | **Attribute level** |
| --- | --- |
| Risk of relapse | 30% less risk |
|  | 50% less risk |
|  | 70% less risk |
| Reducing disease progression | 20% less disease progression |
|  | 40% less disease progression |
|  | 60% less disease progression |
| Risk of side effects | Very common mild side effects (more than 10%  risk) |
|  | Common moderate side effects (1 to 10% risk) |
|  | Rare severe side effects (0.1 to 1% risk) |
| Mode of administration | Injecting treatment once a week |
|  | Injecting treatment 3 times per week |
|  | Taking 1 pill per day orally |
|  | Taking 2 pills per day orally |
|  | Replacing the implant once a year |
|  | Replacing the implant every 3 years |


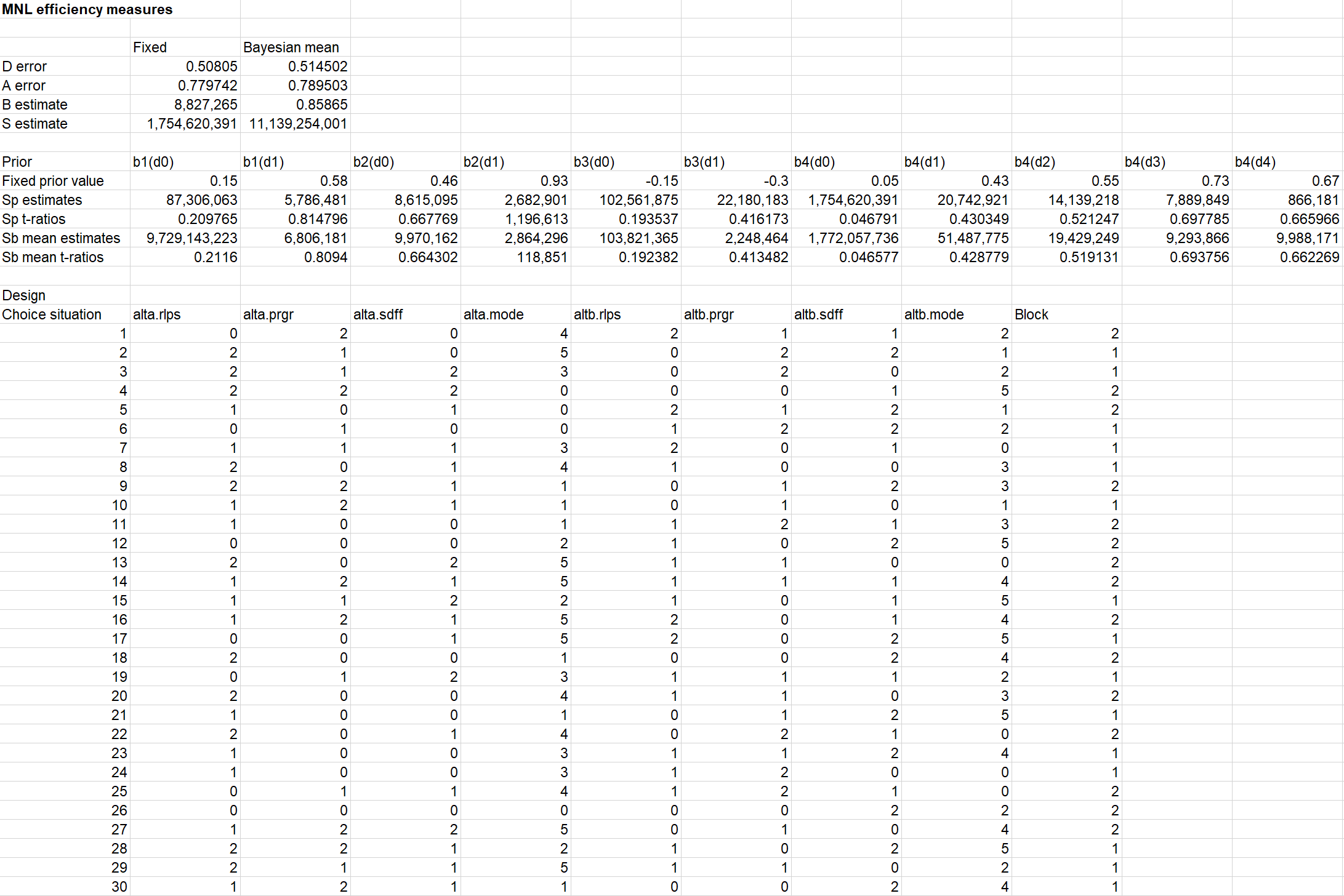


|  |  | **Standard group** | **Choice-matching group** |
| --- | --- | --- | --- |
|  | *Own adherence* | | |
| Been careless about taking your medication? | Median | 4 | 4 |
| Forgotten to take your medication? | Median | 4 | 4 |
| Taken your medication less than your doctor prescribed because you felt better? | Median | 5 | 5 |
| Stopped using your medication because you felt better? | Median | 5 | 5 |
| Sum score | Mean | 4.04  (±0.98) | 4.08  (±0.90) |
|  | Median | 4.25 | 4.25 |
|  | *Others’ adherence* | | |
| Been careless about taking your medication? | Median |  | 5 |
| Forgotten to take your medication? | Median |  | 5 |
| Taken your medication less than your doctor prescribed because you felt better? | Median |  | 5 |
| Stopped using your medication because you felt better? | Median |  | 5 |
| Sum score | Mean |  | 4.54  (±2.76) |
|  | Median |  | 5 |

1. **Mean, median and standard deviations of responses to medication adherence**

*Note: There are no statistically significant differences between the two groups regarding their own medication adherence.*
